# Knockout of the Glucocorticoid Receptor Impairs Reproduction in Female Zebrafish

**DOI:** 10.3390/ijms21239073

**Published:** 2020-11-28

**Authors:** Francesca Maradonna, Giorgia Gioacchini, Valentina Notarstefano, Camilla Maria Fontana, Filippo Citton, Luisa Dalla Valle, Elisabetta Giorgini, Oliana Carnevali

**Affiliations:** 1 Department of Life and Environmental Sciences, Università Politecnica delle Marche, Via Brecce Bianche snc, 60131 Ancona, Italy; f.maradonna@univpm.it (F.M.); giorgia.gioacchini@univpm.it (G.G.); v.notarstefano@univpm.it (V.N.); e.giorgini@univpm.it (E.G.); 2 Biostructures and Biosystems National Institute—Interuniversity Consortium, Viale delle Medaglie d’Oro 305, 00136 Roma, Italy; 3 Department of Biology, Università di Padova, Via Ugo Bassi 58/B, 35131 Padova, Italy; camillamaria.fontana@phd.unipd.it (C.M.F.); filippo.citton@studenti.unipd.it (F.C.)

**Keywords:** glucocorticoids, *gr* mutants, oocyte maturation, vitellogenesis, *Danio rerio*, Fourier transform infrared imaging spectroscopy

## Abstract

The pleiotropic effects of glucocorticoids in metabolic, developmental, immune and stress response processes have been extensively investigated; conversely, their roles in reproduction are still less documented. It is well known that stress or long-lasting therapies can cause a strong increase in these hormones, negatively affecting reproduction. Moreover, the need of glucocorticoid (GC) homeostatic levels is highlighted by the reduced fertility reported in the zebrafish glucocorticoid receptor mutant (*nr3c1^ia30/ia30^*) line (hereafter named *gr^−/−^).* Starting from such evidence, in this study, we have investigated the role of glucocorticoid receptor (Gr) in the reproduction of female zebrafish. Key signals orchestrating the reproductive process at the brain, liver, and ovarian levels were analyzed using a multidisciplinary approach. An impairment of the kiss-GnRH system was observed at the central level in (*gr^−/−^*) mutants as compared to wild-type (*wt*) females while, in the liver, vitellogenin *(vtg)* mRNA transcription was not affected. Changes were instead observed in the ovary, particularly in maturing and fully grown follicles (classes III and IV), as documented by the mRNA levels of signals involved in oocyte maturation and ovulation. Follicles isolated from *gr^−/−^* females displayed a decreased level of signals involved in the acquisition of competence and maturation, causing a reduction in ovulation with respect to *wt* females. Fourier transform infrared imaging (FTIRI) analysis of *gr^−/−^* follicle cytoplasm showed major changes in macromolecule abundance and distribution with a clear alteration of oocyte composition. Finally, differences in the molecular structure of the zona radiata layer of *gr^−/−^* follicles are likely to contribute to the reduced fertilization rate observed in mutants.

## 1. Introduction

Fish reproduction is a complex process regulated by a set of exogenous and endogenous signals, acting along the hypothalamic–pituitary–gonadal (HPG) axis; the hypothalamus delivers specific signals to the pituitary gland, which in turn releases hormones that directly affect the gonads [1].

Several environmental factors [2,3] as well as the occurrence of genetic and epigenetic changes [4,5,6] can interfere with the correct crosstalk along the axis, causing impairment of the reproductive function due to altered gonadal maturation, inhibited ovulation and reduced fecundity. Increasing clues suggest that, in addition to the well-known HPG agents, e.g., kisspeptins, gonadotropin releasing hormones, gonadotropins and sex steroids, glucocorticoids (GCs) are also directly involved in the endocrine control of fish reproduction [7].

While their pleiotropic effects in the regulation of metabolism, development, inflammation, immune and stress responses have been mainly described [8], their roles in reproduction still need further investigation. Basal levels of these steroids in the bloodstream are needed to maintain reproductive functions, while their acute or chronic increases negatively affect gametogenesis, as observed in goat preantral follicle exposed in vitro to cortisol [9]. In mammalian models, the presence of glucocorticoid receptors (GRs) in the uterus is critical for embryo implantation and parturition [8]. They are also required for normal testicular function [10]. Regarding teleosts, the pivotal role of GCs for proper ovarian development has been demonstrated in zebrafish [11]. Cortisol uptake during oogenesis is a dynamic process that changes during follicular development; during vitellogenesis, it accumulates within the ooplasm, playing a crucial role in the hydration and ovulation of mature follicles [11]. In addition, the generation of a zebrafish *gr* mutant line, *nr3c1^ia30^/^ia30^* (*gr^−/−^*), confirmed the GC involvement in reproduction. Though homozygous adults are fertile, they manifest a marked decline in fertility, particularly with advancing age [12]. Compromised egg quality and low embryo viability were also reported in another *gr* zebrafish mutant line, GR^369-^, confirming GC importance in reproduction [13]. This evidence prompted us to investigate the role of GCs and their cognate receptor during reproduction at brain, hepatic and ovarian levels. In the brain, we focused on the GnRH-kisspeptin system, given its upstream role in the regulatory cascade along the reproductive axis. In the liver, we examined vitellogenin *(vtg)* mRNA isoforms, whose proteins are crucial in fish reproduction. In the ovary, we analyzed the expression of a set of genes responsible for oocyte maturation and ovulation in class III b and IV follicles. Data were further integrated with information obtained by Fourier transform infrared imaging (FTIRI) spectroscopy [14,15,16,17,18], to improve the current knowledge on the roles played by GCs and Gr in female zebrafish reproduction.

## 2. Results

### 2.1. Ovarian Histology and Fertility Analysis

**Figure 1:**
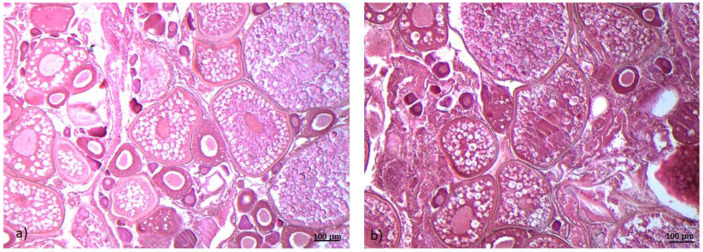
Histological sections of zebrafish ovaries, wild-type *(wt)* (**a**), zebrafish glucocorticoid receptor *(gr^−/−^)* mutant line, *nr3c1^ia30^/^ia30^ (gr^−/−^)* (**b**). Eosin-Gill’s haematoxylin staining (10×). Scale bar 100 um.

Histological analysis showed the presence of all follicular stages in wild-type *(wt)* and *gr^−/−^* ovarian sections. No morphological differences were observed between the experimental groups (Figure 1).

**Figure 2:**
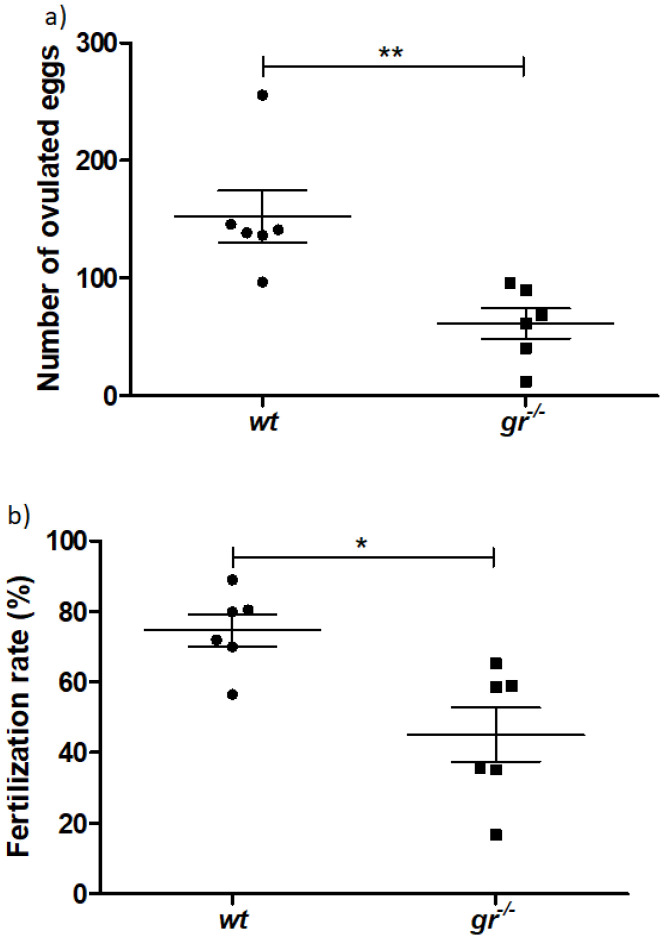
Fish fertility (**a**) number of eggs laid by *wt* females crossed with *wt* males (black circle dots) and *gr^−/−^* females crossed with *wt* males (black square dots); (**b**) fertilization rate in *wt* and *gr^−/−^* females. Asterisks denote statistical significant differences (* *p* < 0.05; ** *p* < 0.01).

However, fertility analysis showed that the number of eggs ovulated by *gr^−/−^* females was less than half of that of *wt* fish (Figure 2a). The fertilization rate in *wt* fish was about 75% and decreased to 45% when *gr^−/−^* females were crossed with *wt* males (Figure 2b); thus, confirming reproductive problems due to the loss of Gr.

### 2.2. Real Time PCR of Signals Involved in Reproduction at Central and Peripheral Levels

With real time PCR, the mRNA expression of KiSS-1 and KiSS-2 metastasis suppressor (*kiss1*, *kiss2*) and gonadotropin releasing hormone 3 (*gnrh3*) was analyzed in the brain of *wt* and mutant fish. *Kiss1* significantly decreased in *gr^−/−^* fish, while no differences were measured regarding *kiss2* and *gnrh3* mRNA levels, (*p* < 0.05) (Table 1a).

In the liver, the mRNA levels of seven vitellogenin (*vtg*) isoforms, *vtg1*, *vtg2*, *vtg3*, *vtg4*, *vtg5*, *vtg6* and *vtg7*, were measured and none of them significantly varied between *wt* and *gr^−/−^* fish (*p* < 0.05) (Table 1b).

Regarding class IIIb isolated follicles, a significant downregulation of progesterone receptor membrane component 1 (*pgrmc1*), *kiss2* and cyclin B1 (*ccnb1*) mRNAs was measured in *gr^−/−^* fish with respect to *wt* ones. Conversely, no difference was found in the expression of mRNAs codifying for the two activin subunits βAa (*inhbaa*) and βB (*inhbb*), growth differentiation factor 9 (*gdf9*), progesterone receptor membrane component 2 (*pgrmc2*), *kiss1*, follicle stimulating hormone receptor (*fshr*), luteinizing hormone/choriogonadotropin receptor (*lhcgr*) and matrix metallopeptidase 9 (*mmp9*), between *gr^−/−^* and *wt* follicles. Concerning class IV isolated follicles, a significant upregulation of *fshr*, and a downregulation of *inhbb*, *pgrmc1*, *kiss1*, *kiss2*, *ccnb1* and *lhcgr* mRNA levels was detected in *gr^−/−^* samples. *Inhbaa*, *pgrmc2* and *mmp9* mRNAs were undetectable in mutant class IV follicles (Table 1c).

### 2.3. Infrared Imaging Analysis

With Fourier transform infrared imaging (FTIRI) spectroscopy, the infrared imaging analysis of small areas of biological samples, such as tissues and cells, was performed. IR maps, in which each pixel corresponds to an IR spectrum and represents the total infrared absorbance in the MIR spectral range (4000–800 cm^−1^) area [19,20], were generated. The integration of IR maps in different spectral ranges, specific for different biocomponents (such as lipids, proteins, and so on), generates false color images showing the topographical distribution of the investigated macromolecules on the mapped area. As for ovarian sections, it was not possible to distinguish between stage IIIa and IIIb oocytes and resulting data refer to class III.

**Figure 3:**
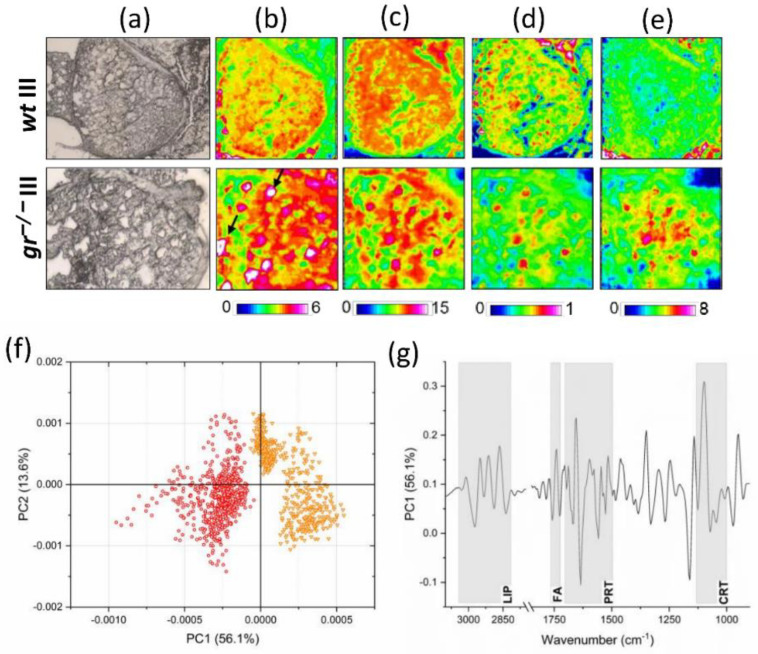
Infrared imaging analysis. (**a**) Microphotographs of class III oocytes in *wt* and *gr^−/−^* ovary sections. False color images showing the topographical distribution of: (**b**) lipids (color scale 0–6), (**c**) proteins (color scale 0–15), (**d**) glycosylated compounds (color scale 0–1), and (**e**) cortisol (color scale 0–8) (the dimensions of false color images were 328 × 328 mm). (**f**) Principal component analysis (PCA) scores’ plot of *wt* III (orange) and *gr^−/−^* (red) spectral data. (**g**) PC1 loadings of *wt* and *gr^−/−^* class III oocytes.

The infrared imaging analysis of *wt* and *gr^−/−^* class III oocytes is reported in Figure 3b–e. A different topographical localization of lipids (Figure 3b, color scale 0–6), proteins (Figure 3c, color scale 0–15), glycosylated compounds (Figure 3d, color scale 0–1), and cortisol (Figure 3e, color scale 0–8) was observed between *wt* and *gr^−/−^* samples. In *wt* III oocytes, lipids, proteins, and glycosylated compounds homogenously colocalize, outlining the presence of yolk vesicles within the ooplasm (Figure 3b–d). Conversely, in *gr^−/−^* III oocytes, lipid distribution suggests the occurrence of several lipid droplets (indicated by black arrows, Figure 3b). Moreover, areas rich in cortisol (Figure 3e) were evidenced in the middle of the oocyte, showing colocalization with proteins (Figure 3c), as confirmed by the presence of carbonyl groups in the cortisol molecule.

To highlight the occurrence of different spectral features, associated with different biochemical composition of the cytoplasm of *wt* and *gr^−/−^* oocytes, the pairwise principal component analysis (PCA) of spectral data was performed. Even though a good segregation of the two spectral populations was found along the PC1 axis (with a variance explained by a PC1 of 56.1%) (Figure 3f), the analysis of the PCA score plot evidenced the presence of not completely homogeneous populations in both *wt* and *gr^−/−^* samples. This result could be caused by the heterogeneous composition of class III samples (IIIa plus IIIb), characterized by slight differences in biochemical composition. The analysis of PC1 loadings suggested differences in the spectral regions related to lipids, fatty acids, proteins, and cortisol (Figure 3g).

To improve the qualitative results obtained by imaging analysis, data were matched with semi-quantitative information from univariate analysis. For this purpose, specific band area ratios meaningful for the biochemical composition of the cytoplasm of *wt* and *gr^−/−^* class III oocytes were calculated (Table 2). With respect to *wt* oocytes, *gr^−/−^* samples were characterized by statistically significant higher amounts of lipids, both in terms of total lipids (LIP/CYT, *p* < 0.01) and saturated lipid alkyl chains (CH2/CYT, *p* < 0.05), and of cortisol (CRT/CYT, *p* < 0.01); conversely, similar levels of glycosylated compounds (COH/CYT, *p* > 0.05) and proteins (PRT/CYT, *p* > 0.05) were found in both experimental groups.

**Figure 4:**
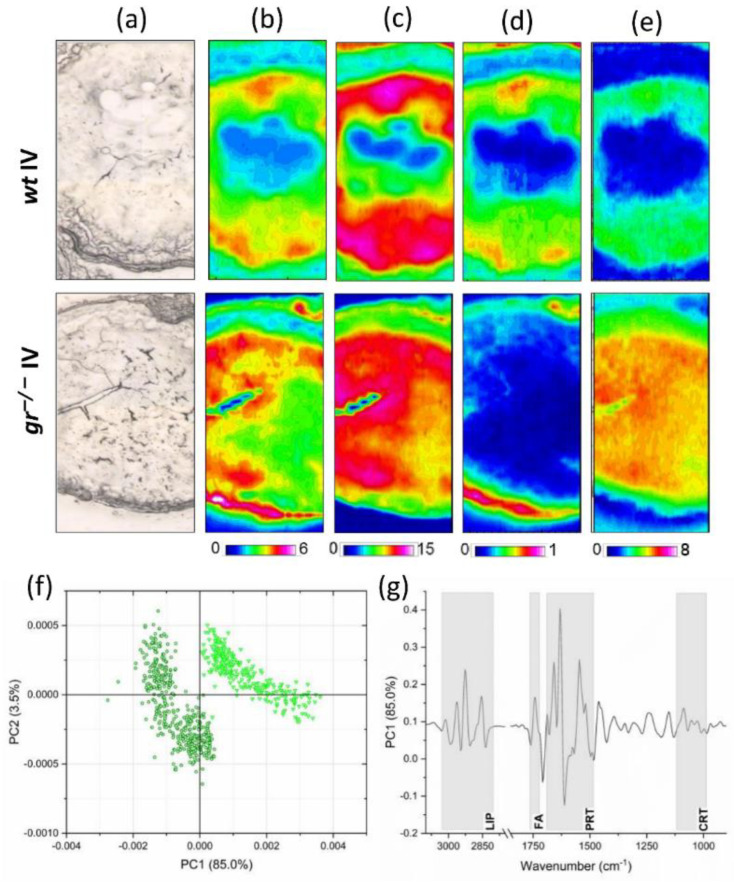
Hyperspectral imaging analysis. (**a**) Microphotographs of class IV oocytes isolated from *wt* and *gr^−/−^* ovaries; false color images showing the topographical distribution of (**b**) lipids (color scale 0–6), (**c**) proteins (color scale 0–15), (**d**) glycosylated compounds (color scale 0–1), and (**e**) cortisol (color scale 0–8) (the dimensions of false color images were 164 × 492 μm). PCA score plots of (**f**) *wt* IV (light green) and *gr^−/−^* IV (dark green) spectral data; PC1 loadings of (**g**) *wt* IV/*gr^−/−^* IV spectral populations.

The same analytical procedure was followed for class IV oocytes. The infrared imaging analysis is reported in Figure 4b–e. The homogeneous distribution of lipids (Figure 4b, color scale 0–6) and proteins (Figure 4c, color scale 0–15) observed in the cytoplasm of both *wt* and *gr^−/−^* class IV oocytes correlates with the process of yolk coalescence. Moreover, *gr^−/−^* samples were characterized by an increase in cytoplasmic cortisol levels (Figure 4e, color scale 0–8) and by a relevant localization of lipids (Figure 4b, color scale 0–6) and glycosylated compounds (Figure 4d, color scale 0–1) in the zona radiata (ZR).

The pairwise PCA analysis of the spectral data from the cytoplasm of *wt* and *gr^−/−^* class IV oocytes showed good segregation between the two experimental groups along the PC1 axis (with a variance with respect to PC1 of 85.0%) (Figure 4f). This result was also confirmed by the analysis of PC1 loadings, which suggested relevant modifications in the whole spectral profiles of *wt* and *gr^−/−^* class IV oocytes (Figure 4g).

From the analysis of the band area ratios (Table 3), the cytoplasm of *gr^−/−^* samples was characterized by a higher amount of total lipids (LIP/CYT, *p* < 0.05), fatty acids (FA/CYT, *p* < 0.01), saturated lipid alkyl chains (CH2/CYT, *p* < 0.01), and cortisol (CRT/CYT, *p* < 0.0001) and similar levels of glycosylated compounds (COH/CYT, *p* > 0.05) and proteins (PRT/CYT, *p* > 0.05).

Since infrared imaging analysis evidenced differences in the distribution of major biomolecules in the ZR of IV oocytes, the specific band area ratios calculated on ZR spectra were statistically analyzed (Table 4).

Results show that ZR was characterized by higher levels of total lipids (LIP/ZR, *p* < 0.05), fatty acids (FA/ZR, *p* < 0.01) and saturated lipid alkyl chains (CH2/ZR, *p* < 0.01), and lower levels of glycosylated compounds (COH/ZR, *p* > 0.0001) and proteins (PRT/ZR, *p* > 0.05).

## 3. Discussion

The present results produce new information on the role played by the glucocorticoid receptor in female zebrafish reproduction and integrate the data already available about the effects of its KO on different physiological processes. Differently from what occurs in *GR^−/−^* mice, which are not viable due to impairment of lung maturation [21], zebrafish mutants reproduce but their reproductive success is reduced and declines with age [12].

At a central level, we found that *gnrh3* mRNA expression is not affected by Gr loss. Although this *gnrh* isoform has been primarily considered a main factor in zebrafish reproduction, recent studies using a *gnrh3* null zebrafish line have shown that the loss of the encoded protein does not impair gonadal development and reproductive capacity, opening an additional field of investigation [22,23]. Moreover, gonadal maturation is not affected by the triple KOs of *gnrh3* and both *kisspeptins 1* isoforms. This suggests the existence of a compensation mechanism able to stimulate the reproductive axis in their absence [22] and likely operative also in our mutants.

In the liver, results show similar levels of *vtg* mRNA isoforms between *wt* and mutants, supporting the integrity of the endocrine system involved in vitellogenesis, a crucial step for oocyte growth of oviparous vertebrates. In these species, *vtg* is synthesized in the liver under estradiol stimulation. Once produced, it is immediately transported and sequestered by the developing oocytes [24,25] to further supply nutrients to the growing embryo. Recent data on zebrafish have reported that *vtg* mRNAs encode for three major types of *vtg* proteins, and their abundance does not change from good to bad quality eggs [26]. Similar *vtg* mRNA levels were observed in our samples but, considering the lower fertility of mutants, we suspect that egg quality may differ between *wt* and *gr^−/−^* follicles. Thus, as already observed in other studies, *vtg* cannot be considered an exclusive biomarker of egg quality.

As for the ovary, the possible causes of the reduced fertility of mutants were examined in class IIIb, competent follicles, and class IV, maturing follicles.

Key factor of oocyte maturation is the 17α,20β-dihydroxyprogesterone (MIH) synthesis by granulosa cells and the oocyte ability to respond to this signal, becoming “maturational competent”. MIH synthesis is triggered by LH [1]. LH acts by binding to its receptor, Lhcgr, whose expression was significantly downregulated in *gr^−/−^* follicles, representing a possible cause of the observed lower fertility. *Lhcgr* expression lags behind that of *fshr* and its levels become detectable when follicles start to accumulate yolk granules, rise steadily afterwards and reach the peak at the full-grown stage before oocyte maturation. So far, *fshr* mRNA levels have not been investigated in class IV follicles [27]. However, previous studies in *wt* zebrafish have reported that *fshr* levels significantly increase as follicles enter vitellogenesis and reach peak in mid-vitellogenic ones. Then, *fshr* levels drop at the full-grown stage, when LH peaks and *lhcgr* is maximally expressed [28]. Previous studies in medaka and mice have demonstrated that an increase in cortisol causes a reduction in *fshr* mRNA/protein levels. In medaka, a rise in temperature determined a whole-body cortisol elevation with subsequent female-to-male sex reversal [29], while in C57BL/6 mice exposed to chronic stress, an increase in corticosterone levels reduced the ovarian follicular reserve and downregulated *fshr* [30]. Therefore, in our *gr^−/−^* mutants, which are both hypercortisolemic and cortisol insensitive, the higher-class IV follicles’ *fshr* levels could be ascribed to the loss of the cortisol negative feedback at the hypothalamic/pituitary axis.

Once synthesized, MIH binds to membrane progesterone receptors (Pgrmcs) on class IIIb oocyte oolemma [27]. Previous data on zebrafish have shown that *pgrmc1* and *2* receptor mRNA levels vary during follicular development, increasing in the most advanced stages [27]. In addition, *pgrmc1^−/−^* zebrafish displayed a reduction in both spawning frequency and in the number of produced embryos [31]. These pieces of evidence, together with the reduction in the *pgrmcs* found herein, suggest the involvement of Gr in the maturation process, possibly through the modulation of progesterone receptors. The reduced sensitivity to progestin is suspected to impair oocyte maturation and ovulation, leading to the observed diminished fertility. Aside from *pgrmcs’* mRNA levels, few mRNA differences were found between *wt* and *gr^−/−^* class IIIb follicles, merely limited to *kiss2* and *ccnb1*. Instead, in class IV follicles, almost all genes analyzed were significantly altered in mutant fish, interfering with LH control of ovulation. Under normal conditions, during the growth phase characterized by vitellogenin uptake, oocytes are blocked at prophase I of meiosis. Prior to ovulation, in class IV follicles, the LH surge stimulates meiosis resumption [22,32]. In *gr^−/−^* class IV follicles, the decrease in *pgrmcs’* mRNA levels is associated with a reduction in *ccnb1* mRNAs, encoding cyclin B, whose temporal control of mRNA translation is important for driving the progression of meiosis [33,34], a step essential to ensure fertility. In vitro *ccnb1* microinjection in fully grown oocytes lacking mPRs activates maturation promoting factor and germinal vesicle breakdown (GVBD) [35].

Recently, a putative role of a peripheral paracrine kisspeptin signaling in the direct control of ovulation was demonstrated in mammals [36]. In the rat ovary, Kiss1 prevents early follicle recruitment by downregulating *fshr* and increasing circulating anti-Müllerian hormone (AMH) levels [37]. It is also involved in the process of oocyte maturation and ovulation [38]. Similar results have been described in pig [39] and sheep [40], where kisspeptin induces in vitro maturation of oocytes by upregulating BMP15, GDF9 and c-MOS levels. Few data are available regarding fish, but the reduction in fertility observed in our female mutant line could be dependent upon the local downregulation of the kiss system.

Activin/inhibin are dimeric proteins of the TGF-β family, controlling several ovarian functions, such as steroidogenesis, granulosa cell proliferation, modulation of *fshr* and follicle development and maturation [41]. Previous studies on zebrafish have shown that Activin βA subunit (codified by *inhbaa* mRNA) levels increase during the growth phase, with a peak in mid-vitellogenic follicles, suggesting a primary role in early follicle recruitment and vitellogenic growth. Instead, the expression of activin βB (*inhbb* mRNA) increases significantly in previtellogenic oocytes, remaining constant from stage IIIa onwards [42]. In the present work, while there was little mRNA variation between *wt* and *gr^−/−^* class IIIb follicles, mutant class IV ones exhibited significantly lower levels of *inhbb* mRNA, suggesting that this condition could also concur in lowering fecundity, directly affecting GVBD and possibly ovulation [42].

Concerning *gdf9*, the similarity of expression in *wt* and mutant follicles of both IIIb and IV classes concurs with a role limited to the earlier stages of follicle recruitment, as previously described [43]. As for the proteinases required for the rupture of ovarian follicles at ovulation [44], the involvement of progestins and progesterone receptor in the control of metalloproteinase activity was demonstrated by the significant reduction in *mmp9* expression in *pgr^−/−^* zebrafish [45]. The fact that, in our mutants, class IV follicles do not express this gene strongly supports a critical role of GCs in the regulation of the ovulation phase.

New insights on oocyte macromolecular composition are provided by FTIRI analysis, used to characterize the biochemical spectral changes associated with oocyte growth and maturation [46] in various species, from fish to humans [46,47,48,49].

FTIRI results show that *gr* KO did not affect vitellogenin uptake, as clearly shown by the band area ratios diagnostic for yolk proteins (PRT/CYT and COH/CYT) [46]; thus, supporting the lack of differences in *vtg* mRNA transcription. Conversely, an increased uptake of lipids, mainly saturated fatty acids (LIP/CYT, FA/CYT and CH2/CYT), in oil droplets within the cytoplasm was observed in mutants. An increase in adipose tissue is a hallmark of *gr* KO [12,50], where cortisol, increased by Gr loss, is diverted to activate the mineralocorticoid receptor that is mainly involved in lipid anabolism, while Gr in catabolism [50]. In maturing *gr^−/−^* oocytes, we have found an accumulation of ooplasmic cortisol, which could differently affect oogenesis, spawning and embryo development [11]. At physiological concentrations, cortisol seems to be marginally involved in GVBD, but variations of its level during oocyte maturation can also affect ovulation, although different outcomes have been described among teleost species [7]. The cortisol increase in *gr^−/−^* follicles recalls that previously measured in *gr^−/−^* mutant mothers [12,51]. Notably, maternal cortisol levels are transferred to the eggs during oogenesis and the complex of this steroid with its cognate receptor, also of maternal origin, participates in the maternal programming of embryo development [52,53]. The lack of Gr receptor, however, is not incompatible with zebrafish offspring survival and reproduction.

Regarding ZR composition, its glycoproteins have the primary role of leading a single spermatozoa into the micropyle ensuring the successful egg fertilization [54]. Thus, the alteration of glycosylate compounds and protein abundance observed for the first time in the ZR of *gr^−/−^* mutants may provide a rationale for their reduced fertilization rate.

In conclusion, our results clearly confirm the critical role of GCs and their cognate receptor in fish reproduction. Gr ablation adversely affects reproduction by causing changes in ooplasm composition and altering the signaling pathways responsible for ovarian maturation and ovulation.

## 4. Materials and Methods

### 4.1. Zebrafish Maintenance and Oocyte Isolation

Six month-old wild type (*wt* control group) and mutant *nr3c1^ia30/ia30^* (*gr^−/−^* experimental group) zebrafish were maintained according to standard procedures, as previously described [12]. The genetic background of the *wt* fish was the same used for the generation of the *gr* mutant line. All husbandry and experimental protocols complied with the European Legislation for the Protection of Animals used for Scientific Purposes (Directive 2010/63/EU), and were previously authorized by the Ministero della salute, (Number 112/2015-PR). Fifteen *wt* and fifteen *gr^−/−^* females were sacrificed using a lethal overdose of anesthetic (500 mg/L of 3-aminobenzoic acid ethyl ester (MS-222) buffered at pH 7.4; Sigma, Milan, Italy). Brain and liver samples were stored at −80 °C for biomolecular analysis. Ovaries were dissected out and divided as follows: for each group, 5 samples were stored at −80 °C for FTIRI analysis and 5 samples were fixed in Bouin’s fixative for histology. The remaining 5 ovaries were teased into separate follicles using transfer pipettes (Semco Scientific Corp., San Diego, CA, USA) without trypsinization; thereafter, follicles were separated into different maturation stages according to their diameters, as previously described [55] and class IIIb and IV follicles were collected and stored at −80 °C for molecular analysis.

### 4.2. Histological Analysis

Intact gonadal tissues from *wt* and *gr^−/−^* females were carefully dissected from the euthanized fish and fixed in buffered Bouin’s fixative overnight, dehydrated and embedded in Paraplast. Sections of 7 μm thickness were stained with hematoxylin and eosin (H&E). The staging of ovarian follicles was based on previously described classification [55,56].

### 4.3. Fish Fertility

Reproductive performances of six *gr^−/−^* and *wt* female fishes were assayed in spawning tanks under standard conditions. *wt* males were pre-screened by testing their capacity to mate. *wt* and *gr^−/−^* females were transferred in breeding tanks and crossed with *wt* males at the ratio of 1:1. The number of ovulated eggs per clutch and the fertility rate (fertility = fertilized eggs/total eggs × 100%) [57] were assessed. Each female was tested seven times with 7–10 days of interval.

### 4.4. RNA Extraction and cDNA Synthesis

Total RNA was extracted from 5 *wt* and 5 *gr^−/−^* livers and brains and from 3 pools, containing 50 follicles each, of classes IIIb and IV (3 *wt* and 3 *gr^−/−^*) using RNAeasy^®^ Minikit (Qiagen, Milano, Italy). RNA quality assessment and cDNA synthesis were performed as previously described [58,59].

### 4.5. Real-Time PCR

qRT-PCRs were performed with SYBR green in an CFX thermal cycler, as previously described [16]. Ribosomal protein 13 (*rpl13*) and ribosomal protein 0 (*rplp0*) mRNAs were used as internal standards in each sample in order to standardize the results by eliminating variation in mRNA and cDNA quantity and quality. Primer sequences, GenBank accession numbers and primer efficiency of the examined genes are reported in Table 5.

Data were analyzed using iQ5 Optical System version 2.1 (Bio-Rad, Hercules, CA, USA), including Genex Macro iQ5 Conversion and Genex Macro iQ5 files. Modification of gene expression among the experimental groups is reported as relative mRNA abundance (arbitrary units). Primers were used at a final concentration of 10 pmol/mL.

### 4.6. Fourier Transform Infrared Imaging (FTIRI) Measurements and Data Analysis

FTIRI measurements were carried out at the Infrared Beamline Synchrotron Infrared Source for Spectroscopy and Imaging (SISSI), Elettra Sincrotrone Trieste (Trieste, Italy). A Bruker VERTEX 70 interferometer coupled with a Hyperion 3000 Vis-IR microscope was used. The spectrometer was equipped with a liquid nitrogen-cooled bidimensional focal plane array (FPA) detector.

From each frozen ovarian sample, three thin sections (∼10 μm thickness) were cut, 2 mm from each other, by using a cryomicrotome. Sections were immediately deposited, without any fixation process, onto CaF2 optical windows (1 mm thickness, 13 mm diameter) and left to air-dry for 30 min. FTIRI analysis of all samples was performed within 48 h after cutting; this procedure, previously validated, guarantees a good stability in terms of infrared features [62].

For each section, three oocytes of classes III and IV were selected; it was not possible to distinguish between IIIa and IIIb since the analysis was performed on ovarian sections, instead of single mechanically isolated oocytes [19].

The IR maps of these selected oocytes were acquired in transmission mode in the 4000–800 cm^−1^ spectral range and with a spectral resolution of 4 cm^−1^. The mapped areas were 328 × 328 μm size and 164 × 492 μm size for class III and class IV samples, respectively; a constant spatial resolution of 2.56 × 2.56 μm was applied. Raw IR maps were corrected to avoid the interference of water vapor and then vector normalized to minimize differences in sections’ thickness (Atmospheric Compensation and Vector Normalization routines, OPUS 7.1, Bruker, Ettlingen, Germany).

On these pre-processed IR maps, the infrared imaging analysis was carried out. False color images were generated, representative of the topographical distribution of lipids (3050–2800 cm^−1^), proteins (1700–1480 cm^−1^), glycosylated compounds (1188–1137 cm^−1^) and cortisol (1140–1006 cm^−1^). The biochemical assignment of the spectral signals was completed according to the literature [23,24]. Due to the distinct ability of biomolecules to absorb the infrared radiation, different color scales were used (lipids, color scale 0–6; proteins, color scale 0–15; glycosylated compounds, color scale 0–1, and cortisol, color scale 0–8); in any case, the white color represents the highest absorption values, while the blue color represents the lowest ones.

Forty spectra were extracted from the cytoplasm compartment of each mapped oocyte. These selected spectra were interpolated into the 3050–900 cm^−1^ spectral range, two-point-baseline fitted, vector normalized (pre-processed spectra named hereafter) and then submitted to pairwise principal component analysis (PCA) (*wt* III vs. *gr^−/−^* III and *wt* IV vs. *gr^−/−^* IV); PC scores and loadings were also considered (OriginPro 2018b software, OriginLab Corporation, Northampton, MA, USA).

On the same pre-processed spectra, the integrated areas of the following spectral ranges were calculated: 3002–2820 cm^−1^ (stretching modes of CH2 and CH3 groups in lipid alkyl chains; LIP); 1763–1717 cm^−1^ (stretching mode of C = O groups in fatty acids; FA); 1717–1486 cm^−1^ (amide I and II bands of proteins; PRT); 1486–1429 cm^−1^ (bending modes of CH2 groups in lipid alkyl chains; CH2); 1188–1137 cm^−1^ (stretching modes of COH moieties in glycosylated compounds; COH), and 1137–1000 cm^−1^ (stretching of C-O groups in cortisol; CRT) [23,24]. Specific band area ratios were calculated: LIP/CYT, FA/CYT, CH2/CYT, PRT/CYT, COH/CYT, and CRT/CYT. CYT, calculated as the sum of the integrated areas of the spectral ranges 3002–2820 cm^−1^ and 1763–900 cm^−1^, can be considered representative of the total biomass of oocyte cytoplasm.

Finally, ~10 spectra were extracted from the zona radiata (ZR) of all mapped oocytes of class IV. Spectra were submitted to the same treatments of pre-processing and integration discussed above. The following band area ratios were analyzed: LIP/ZR, FA/ZR, CH2/ZR, PRT/ZR, and COH/ZR. The calculated sum of the integrated areas of the spectral ranges 3002–2820 cm^−1^ and 1763–900 cm^−1^ can be considered representative of the total biomass of the ZR of class IV oocytes.

### 4.7. Statistical Analysis

Real time PCR results are presented as arbitrary units means ± S.D. Two-way ANOVA followed by the Tukey test as a multiple comparisons test was used to compare mRNA levels among *wt* and *gr^−/−^* class IIIb and IV follicles. Significance of the PCR results in brains and livers was determined by Student’s *t*-tests. Normally distributed data derived from IR analysis were reported as arbitrary units and presented as means ± S.D. Significant differences between experimental groups were determined by Student’s *t*-tests. Letters indicate statistically significant differences between *wt* and *gr^−/−^* experimental groups, with significance accepted at *p* < 0.05. Fertility data are presented as means ± S.D and were determined by Student’s *t*-tests.

Asterisks indicate statistically significant differences between *wt* and *gr^−/−^* experimental groups (* *p* < 0.05; ** *p* < 0.01). All statistical analyses were performed using the statistical software package Prism6 (GraphPad Software, San Diego, CA, USA).

## Figures and Tables

**Table 1:** (**a**) KiSS-1 metastasis suppressor (*kiss1),* kisspeptin 2 (*kiss2),* gonadotropin releasing factor 3 *(gnrh3)* mRNA values normalized against ribosomal protein 0 (*rplp0)* and ribosomal protein 13 (*rpl13)* in the brain of *wt* and *gr^−/−^* females. (**b**) Vitellogenin *(vtg)* mRNA values normalized against *rplp0* and *rpl13* in the liver of *wt* and *gr^−/−^* female. (**c**) βAa *(inhbaa),* βB (*inhbb),* growth differentiation factor 9 (*gdf9),* progesterone receptor membrane component 1 *(pgrmc1),* progesterone receptor membrane component 2 (*pgrmc2), kiss1, kiss2,* cyclin B1 *(ccnb1),* follicle stimulating hormone receptor (*fshr),* luteinizing hormone/choriogonadotropin receptor *(lhcgr),* matrix metallopeptidase 9 *(mmp9)* mRNA values normalized against *rplp0* and *rpl13* in class IIIb and IV follicles isolated from *wt* and *gr^−/−^* ovaries. Values are presented as arbitrary units (a.u.) and are expressed as means ± S.D. Different letters denote significant differences among experimental groups (*p* < 0.05).

(**a**)				
**Brain-mRNA Expression (a.u.)**	***wt***		***gr^−/−^***	
*kiss 1*	11.76 ± 2.13 ^a^		3.87 ± 2.94 ^b^	
*kiss 2*	1.81 ± 0.19 ^a^		1.39 ± 0.57 ^a^	
*gnrh3*	17.41 ± 9.84 ^a^		14.06 ± 14.76 ^a^	
(**b**)				
**Liver-mRNA Expression (a.u.)**	***wt***		***gr^−/−^***	
*vtg1*	2.42 ± 0.45 ^a^		2.28 ± 2.07 ^a^	
*vtg2*	1.61 ± 0.59 ^a^		2.31 ± 1.75 ^a^	
*vtg3*	1.61 ± 0.60 ^a^		6.64 ± 4.58 ^a^	
*vtg4*	1.58 ± 0.59 ^a^		2.06 ± 1.11 ^a^	
*vtg5*	1.57 ± 0.32 ^a^		2.87 ± 1.98 ^a^	
*vtg6*	1.12± 0.28 ^a^		1.17 ± 0.98 ^a^	
*vtg7*	1.57 ± 0.87 ^a^		2.06 ± 1.11 ^a^	
(**c**)				
**Follicle-mRNA Expression (a.u)**	**III b**		**IV**	
	***wt***	***gr*** *^−/−^*	***wt***	***gr*** *^−/−^*
*inhbaa*	9.24 ± 0.74 ^a^	8.32 ± 0.5 ^a^	5.3 ± 0.14 ^b^	n.d
*inhbb*	4.39 ± 0.27 ^a^	3.06 ± 1.69 ^a^	6.4 ± 1.27 ^b^	1.37 ± 0.59 ^a^
*gdf9*	1.78 ± 0.61 ^a^	1.20± 0.15 ^a^	1.40 ±0.56 ^a^	1.42 ± 0.60 ^a^
*pgrmc1*	7.27 ± 0.61 ^a^	1.48 ± 0.45 ^b^	35.45 ± 2.57 c	22.93 ± 2.93 d
*pgrmc2*	5.33 ± 3.78 ^a^	12.45 ± 4.21 ^a^	38.34 ± 2.83 ^b^	n.d.
*kiss1*	3.23 ± 0.22 ^a^	4.67 ± 0.17 ^a^	35.0 ± 4.24 ^b^	2.24 ± 0.36 ^a^
*kiss2*	27.238 ± 11.27 ^a^	8.42 ± 4.71 ^b^	27.27 ± 0.38 ^a^	4.75 ± 3.18 ^b^
*ccnb1*	8.47 ± 0.26 ^a^	5.58 ± 0.81 ^b^	7.47 ± 0.55 ^a^	1.1 ± 0.14 ^c^
*fshr*	1.95 ± 1.16 ^a^	3.13 ± 0.69 ^a^	2.81 ± 0.43 ^a^	18.19 ± 0.27 ^b^
*lhcgr*	1.09 ± 0.09 ^a^	1.48 ± 0.30 ^a^	31.31 ± 2.16 ^b^	21.49 ± 3.18 ^c^
*mmp9*	1.87 ± 0.92 ^a^	2.17 ± 0.24 ^a^	5.18 ±1.89 ^b^	n.d.

**Table 2:** Biochemical composition of the cytoplasm of class III *wt* and *gr^−/−^* oocytes.

	*wt* III	*gr^−/−^* III
LIP/CYT	0.060 ± 0.013 ^a^	0.099 ± 0.012 ^b^
CH2CYT	0.0050 ± 0.0011 ^a^	0.0069 ± 0.0010 ^b^
PRT/CYT	0.40 ± 0.012 ^a^	0.39 ± 0.030 ^a^
COH/CYT	0.00082 ± 0.00011 ^a^	0.00097 ± 0.00012 ^a^
CRT/CYT	0.106 ± 0.019 ^a^	0.144 ± 0.021 ^b^

Statistical analysis of meaningful band area ratios calculated from IR data: LIP/CYT, relative amount of total lipids; CH2/CYT relative amount of saturated lipid alkyl chains; PRT/CYT, relative amount of proteins; COH/CYT, relative amount of glycosylated compounds, and CTR/CYT, relative amount of cortisol. Data are expressed as arbitrary units and presented as mean ± S.D. Different letters indicate statistically significant differences (*p* < 0.05, Student’s *t*-tests; Prism6, GraphPad Software, San Diego, CA, USA).

**Table 3:** Biochemical composition of the cytoplasm of class IV *wt* and *gr^−/−^* oocytes.

	*wt* IV	*gr^−/−^* IV
LIP/CYT	0.091 ± 0.005 ^a^	0.112 ± 0.006 ^b^
CH2CYT	0.0090 ± 0.0007 ^a^	0.0106 ± 0.0005 ^b^
FA/CYT	0.0061 ± 0.0006 ^a^	0.0075 ± 0.0006 ^b^
PRT/CYT	0.45 ± 0.011 ^a^	0.43 ± 0.016 ^a^
COH/CYT	0.00123 ± 0.00011 ^a^	0.00137 ± 0.00012 ^a^
CRT/CYT	0.034 ± 0.0024 ^a^	0.042 ± 0.0024 ^b^

Statistical analysis of meaningful band area ratios calculated from IR data: LIP/CYT, relative amount of total lipids; CH2/CYT relative amount of saturated lipid alkyl chains; FA/CYT, relative amount of fatty acids; PRT/CYT, relative amount of proteins; COH/CYT, relative amount of glycosylated compounds, and CTR/CYT, relative amount of cortisol. Data are expressed as arbitrary units and presented as means ± S.D. Different letters indicate statistically significant differences (*p* < 0.05, Student’s *t*-tests; Prism6, GraphPad Software, San Diego, CA, USA).

**Table 4:** Biochemical composition of the zona radiata of class IV *wt* and *gr^−/−^* oocytes.

	*wt* ZR	*gr^−/−^* ZR
LIP/ZR	0.058 ± 0.008 ^a^	0.127 ± 0.022 ^b^
CH2/ZR	0.0049 ± 0.0009 ^a^	0.0092 ± 0.0016 ^b^
FA/ZR	0.0038 ± 0.0009 ^a^	0.0054 ± 0.0005 ^b^
PRT/ZR	0.34 ± 0.024 ^a^	0.18 ± 0.020 ^b^
COH/ZR	0.101 ± 0.014 ^a^	0.200 ± 0.051 ^b^

Statistical analysis of meaningful band area ratios calculated from IR data: LIP/ZR, relative amount of total lipids; CH2/ZR relative amount of saturated lipid alkyl chains; FA/ZR, relative amount of fatty acids; PRT/ZR, relative amount of proteins, and COH/ZR, relative amount of glycosylated compounds. Data are expressed as arbitrary units and presented as means ±S.D. Different letters indicate statistically significant differences (*p* < 0.05, Student’s *t*-tests; Prism6, GraphPad Software, San Diego, CA USA).

**Table 5:** Primer sequences, GenBank accession numbers, primer efficiency of the examined genes and source.

Gene	For Sequence 5′-3′	Rew Sequence 5′-3′	Accession Number	Primer Efficiency	Source
*inhbaa*	TGCTGCAAGCGACAATTTTA	CATTCGTTTCGGGACTCAAG	AF475092	87%	[42]
*inhbb*	CAACTTAGATGGACACGCTG	GTGGATGTCGAGGTCTTGTC	X76051	85%	[42]
*fshr*	GATTCTTCACCGTCTTCTCC	TGTAGCTGCTCAACTCAAACA	NM001001812.1	92%	[57]
*gdf9*	CGACCACAACCACCTCTCTCC	GGGACTGAGTGCTGGTGGATGCC	NM001012383.1	95%	
*lhgcr*	GGCGAAGGCTAGATGGCACAT	TCGCAATCTGGTTCATCAATA	NM_205625.1	98%	
*pgrmc1*	CGGTTGTGATGGAGCAGATT	AGTAGCGCCAGTTCTGGTCA	NM_001007392.1	87%	
*pgrmc2*	ACAACGAGCTGCTGAATGTG	ATGGGCCAGTTCAGAGTGAG	NM_213104.1	87%	
*kiss1*	ACAGACACTCGTCCCACAGATG-	CAATCGTGTGAGCATGTCCTG	NM001113489.1	90%	[60]
*kiss2*	ATTCTCTTCATGTCTGCAATGGTCA	TGCTTCTCAGGTAAAGCATCATTG	NM001142585.1	93%	
*ccnb1*	GAAATGATGGCTCTCCGTGT	ACCACAGGTGCCTTCTCAAC	NM131513	80%	This paper
*gnrh3*	TTAGCATGGAGTGAAAAGGAAGGTTG	ACCACAGGTGCCTTCTCAAC	NM001177450.1	82%	[60]
*mmp9*	CATTAAAGATGCCCTGATGTATCCC	AGTGGTGGTCCGTGGTTGAG	NM213123	*85%*	[12]
*vtg1*	GATTAAGCGTACACTGAGACCA	AGCCACTTCTTGTCCAAATACT	NM001044897	90%	[61]
*vtg2*	TGCCGCATGAAACTTGAATCT	GTTCTTACTGGTGCACAGCC	NM001044913	91%	
*vtg3*	GGGAAAGGATTCAAGATGTTCAGA	ATTTGCTGATTTCAACTGGGAGAC	NM131265	99%	
*vtg4*	TCCAGACGGTACTTTCACCA	CTGACAGTTCTGCATCAACACA	NM001045294	87%	
*vtg5*	ATTGCCAAGAAAGAGCCCAA	TTCAGCCTCAAACAGCACAA	NM001025189	91%	
*vtg6*	TTTGGTGTGAGAACTGGAGG	CCAGTTTGTGAGTGCTTTCAG	NM001122610	89%	
*vtg7*	TTGGTGTGAGAACTGGAGGA	TTGCAAGTGCCTTCAGTGTA	NM001102671	93%	
*rplp0*	CTGAACATCTCGCCCTTCTC	TAGCCGATCTGCAGACACAC	NM131580.1	98%	[57]
*rpl13*	TCTGGAGGACTGTAAGAGGTATGC	AGACGCACAATCTTGAGAGCAG	NM_212784.1	98%	[16]
